# Metabolic syndrome criteria and severity and carbon dioxide (CO_2_) emissions in an adult population

**DOI:** 10.1186/s12992-023-00948-3

**Published:** 2023-07-13

**Authors:** Silvia García, Rosario Pastor, Margalida Monserrat-Mesquida, Laura Álvarez-Álvarez, María Rubín-García, Miguel Ángel Martínez-González, Jordi Salas-Salvadó, Dolores Corella, Albert Goday, J. Alfredo Martínez, Ángel M. Alonso-Gómez, Julia Wärnberg, Jesús Vioque, Dora Romaguera, José Lopez-Miranda, Ramon Estruch, Francisco J. Tinahones, José Lapetra, Lluís Serra-Majem, Blanca Riquelme-Gallego, Xavier Pintó, José J. Gaforio, Pilar Matía, Josep Vidal, Clotilde Vázquez, Lidia Daimiel, Emilio Ros, Carmen Sayón-Orea, Patricia Guillem-Saiz, Cristina Valle-Hita, Robert Cabanes, Itziar Abete, Leire Goicolea-Güemez, Enrique Gómez-Gracia, Cristina Tercero-Maciá, Antoni Colom, Antonio García-Ríos, Sara Castro-Barquero, José C. Fernández-García, José Manuel Santos-Lozano, Juan Carlos Cenoz, Rocío Barragán, Nadine Khoury, Olga Castañer, María Ángeles Zulet, Jessica Vaquero-Luna, Maira Bes-Rastrollo, Sara de las Heras-Delgado, Ramon Ciurana, Vicente Martín-Sánchez, Josep A. Tur, Cristina Bouzas

**Affiliations:** 1 CIBER Fisiopatología de la Obesidad y Nutrición (CIBEROBN), Instituto de Salud Carlos III (ISCIII), Madrid, 28029 Spain; 2 Research Group on Community Nutrition & Oxidative Stress, University of the Balearic Islands & CIBEROBN, Guillem Colom Bldg, Campus, E-07122 Palma de Mallorca, Spain; 3 Health Research Institute of the Balearic Islands (IdISBa), Palma de Mallorca, 07120 Spain; 4 Faculty of Health Sciences, Catholic University of Avila, Avila, 05005 Spain; 5 CIBER Epidemiología y Salud Pública (CIBERESP), Instituto de Salud Carlos III (ISCIII), Madrid, 28029 Spain; 6 Institute of Biomedicine (IBIOMED), University of León, Leon, 24071 Spain; 7 Department of Preventive Medicine and Public Health, University of Navarra, IDISNA, Pamplona, 31008 Spain; 8 Biochemistry and Biotechnology Department. Human Nutrition Unit, Universitat Rovira i Virgili, IISPV, Hospital Universitari de Sant Joan, Reus, 43201 Spain; 9 Department of Preventive Medicine, University of Valencia, Valencia, 46100 Spain; 10 Unit of Cardiovascular Risk and Nutrition, Institut Hospital del Mar de Investigaciones Médicas Municipal d’Investigació Mèdica (IMIM), Barcelona, 08003 Spain; 11 Departament of Medicine, Universitat Autònoma de Barcelona, Barcelona, 08003 Spain; 12 Cardiometabolics Precision Nutrition Program, IMDEA Food, CEI UAM + CSIC, Madrid, 28049 Spain; 13 Department of Nutrition, Food Sciences, and Physiology, Center for Nutrition Research, University of Navarra, Pamplona, 31008 Spain; 14 Osakidetza Basque Health Service, Bioaraba Health Research Institute, Araba University Hospital, University of the Basque Country UPV/EHU, Vitoria-Gasteiz, 48013 Spain; 15 Department of Nursing, School of Health Sciences, University of Málaga-IBIMA, Málaga, 29071 Spain; 16 Instituto de Investigación Sanitaria y Biomédica de Alicante, Universidad Miguel Hernández (ISABIAL-UMH), Alicante, 03550 Spain; 17 Lipids and Atherosclerosis Unit, Department of Internal Medicine, Reina Sofia University Hospital, Maimonides Biomedical Research Institute of Cordoba (IMIBIC), University of Cordoba, Córdoba, 14004 Spain; 18 Department of Internal Medicine, IDIBAPS, Hospital Clinic, University of Barcelona, Barcelona, 08036 Spain; 19 Department of Endocrinology, Virgen de la Victoria Hospital, University of Málaga, Málaga, 29010 Spain; 20 Department of Family Medicine, Research Unit, Distrito Sanitario Atención Primaria Sevilla, Sevilla, 41013 Spain; 21 Institute for Biomedical Research, University of Las Palmas de Gran Canaria, Las Palmas, 35016 Spain; 22 Department of Preventive Medicine, University of Granada, Granada, 18071 Spain; 23 Lipids and Vascular Risk Unit, Internal Medicine, Hospital Universitario de Bellvitge, Hospitalet de Llobregat, Barcelona, 08907 Spain; 24 Department of Health Sciences, Center for Advanced Studies in Olive Grove and Olive Oils, University of Jaen, Jaen, 23071 Spain; 25 Department of Endocrinology and Nutrition, Instituto de Investigación Sanitaria San Carlos (IdISSC), Madrid, 28040 Spain; 26 Department of Endocrinology, IDIBAPS, Hospital Clinic, University of Barcelona, Barcelona, 08036 Spain; 27 Department of Endocrinology, Fundación Jiménez-Díaz, Madrid, 28040 Spain; 28 Precision Nutrition and Obesity Program.IMDEA Food, Nutritional Control of the Epigenome Group, CEI UAM + CSIC, Madrid, 28049 Spain; 29 Department of Endocrinology and Nutrition, Lipid Clinic, Institut d’Investigacions Biomèdiques August Pi Sunyer (IDIBAPS), Hospital Clínic, Barcelona, 08036 Spain; 30 Navarra Institute of Public Health. Regional Health Service of Navarra, Pamplona, Spain; 31 Department of Public Health and Psychiatry, School of Medicine, Biomedical Research Institute of Malaga (IBIMA), University of Malaga, Málaga, 29010 Spain; 32 Centro Salud Raval, Elche-Alicante, 03203 Spain; 33 Navarra Regional Health Service, Primary Health Care Services, Pamplona, Spain

**Keywords:** Metabolic syndrome, Environment, CO_2_ emissions, Non-communicable diseases, Glycaemia, Diet

## Abstract

**Background:**

Metabolic syndrome (MetS) has become a growing risk factor of some non-communicable diseases. Increase of greenhouse gas emissions affects the planet.

**Aims:**

To assess the association between MetS severity and amount of carbon dioxide (CO_2_) emitted in an adult population.

**Design:**

Cross-sectional study (*n* = 6646; 55-76-year-old-men; 60-75-year-old-women with MetS).

**Methods:**

Dietary habits were assessed using a pre-validated semi quantitative 143-item food frequency questionnaire. The amount of CO_2_ emitted due to the production of food consumed by person and day was calculated using a European database, and the severity of the MetS was calculated with the MetS Severity Score.

**Results:**

Higher glycaemia levels were found in people with higher CO_2_ emissions. The risk of having high severe MetS was related to high CO_2_ emissions.

**Conclusions:**

Low CO_2_ emissions diet would help to reduce MetS severity. Advantages for both health and the environment were found following a more sustainable diet.

**Trial registration:**

ISRCTN, ISRCTN89898870. Registered 05 September 2013.

## Introduction

Several factors have a detrimental impact on the ecosystem, including the amount of greenhouse gas emissions (GHGs) in the atmosphere, especially carbon dioxide (CO_2_) [1]. The intergovernmental Panel on Climate Change (IPCC) aimed to reduce emissions by 45% by 2030 and achieve zero emissions by 2050 [2]. There is a vital need for a more in-depth evaluation to assess the impact of health and related factors on climate change, and vice versa, considering the different scenarios of climate change and predictions of the demographic structure of the countries [1]. Food system emissions are around one third of the global GHG emissions and represents 34% of total CO_2_ equivalents in 2015 [3]. Therefore, food production and dietary consumption are related to climate change, and they should be changed [4] to reduce emissions caused from it.

Metabolic syndrome (MetS) is a condition of interrelated risk factors including high glycaemia levels (> 100 mg/dL), hypertension (> 130/85 mmHg), raised triglyceride levels (> 150 mg/dL), low high-density lipoprotein cholesterol levels (< 40 mg/dL in men; <50 mg/dL in women), and abdominal obesity (waist circumference of > 102 cm in men; >88 cm in women) [5–7]. Three or more of these factors are considered as having MetS and lead to increased risk of cardiovascular disease, atherosclerosis, cancer, and type 2 diabetes mellitus (T2DM) [8] which are some of the main causes of death in Spain [9] but also worldwide [10]. The severity of having one or more aggregated cardiovascular risk factors for metabolic syndrome is known as the metabolic syndrome severity (MetSS), and can be measured with the metabolic syndrome severity score (MetSSS). This is a tool created to assess cardio-metabolic risk considering the joint effect of metabolic syndrome factors, and not their separated presence. It can be used for the treatment and management of this pathology [11].

MetS is closely interrelated with food habits. A high consumption of high calorie and low-density food and a reduction of physical activity (PA) has led to an increase of MetS in Western countries but also in developing countries which have changed to a Western lifestyle [12]. Moderate or vigorous exercise has been inversely associated with MetS in children and adolescent population, while a sedentary behavior has been positively associated [13, 14], and the same effects have been observed in adult population [15–17]. Dietary habits [17, 18] and weight interventions [19] have been related to MetS. Different diet interventions such as time-restricted eating [20] or high-protein diet [21] has been studied, but the Mediterranean diet (MedDiet) seemed to be one of the most effective interventions [14, 17, 22–25]. MetS is also influenced and increases with age [18, 26]. Its prevalence is 31% in Spain [27], and it increasingly becoming a global epidemic [11, 12].

Since food production and dietary consumption are related to climate change and globalization-related drivers of MetS are related to climate change, it could be inferred that MetS could be related to environmental change. The risk of MetS is increasing and reaching epidemic proportions worldwide [28] and a detrimental impact on the environment is also happening [29]; then, the dietary intake or food consumption could be part of the problem.

The risk of MetS and diet-related CO_2_ emissions should be both attenuated changing food habits and following certain diets such as the MedDiet which is a well-studied model in terms of healthiness and sustainability [30, 31]. Sustainable diets were defined as those diets with low environmental impacts which contribute to food and nutrition security and to healthy life for present and future generations. Sustainable diets are protective and respectful of biodiversity and ecosystems, culturally acceptable, accessible, economically fair and affordable; nutritionally adequate, safe and healthy; while optimizing natural and human resources [32]. Few papers linking MetS and sustainability, or GHGs, have been found.

Therefore, the aim of this study was to assess the association between the MetS severity, and the amount of CO_2_ emitted from the production of food consumed, in an adult population.

## Methods

### Study design

The current research was a cross-sectional analysis of the baseline data within an ongoing 8-year multicenter, parallel-group, randomized trial, carried out in 23 Spanish recruitment centers. More details of the study design have been extensively described [33]. The trial was registered in 09/05/2013 at the International Standard Randomized Controlled Trial (ISRCT; http://www.isrctn.com/ISRCTN89898870) with the number 89898870.

#### Participants, recruitment, randomization, and ethics

**Fig. 1:**
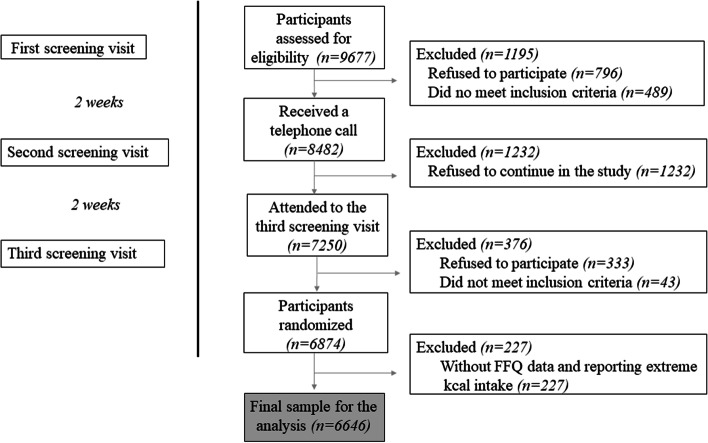
Flow chart of participant inclusion

Participant flow-chart eligibility was shown in Fig. 1. Among the 9677 participants who were contacted, 6874 participants met the inclusion criteria of being men aged 55–76 or women aged 60–75, overweight or obese (body mass index (BMI) between 27 and 40 kg/m^2^) and meeting at least three criteria for the MetS according to the Association and National Heart, Lung, and Blood Institute [5]. Participants with incomplete food frequency questionnaire (FFQ) data or reporting extreme total energy intakes (< 500 or > 3500 kcal/day in women or < 800 or > 4000 kcal/day in men) were excluded and 6646 participants were left for the analysis. Informed written consent was provided by all participants and the study protocol and procedures were approved by ethical committees according to the ethical standards of the Declaration of Helsinki by all the 23 participating institutions.

#### Dietary assessment

Registered dietitians assessed dietary habits, at baseline, using a semi quantitative 143-item FFQ [34] previously validated in the Spanish population [35, 36]. A regular portion size was established for each item, and 9 categories (ranging from “never or almost never” to “≥6 times/day”) were used to assess consumption frequencies. Energy and nutrient intakes were calculated as frequency multiplied by nutrient composition of specified portion size for each food item, using a computer program based on available information in Spanish food composition Tables [37, 38]. The results were used to determine the specific amount of food (in grams) each participant had eaten per day [39, 40].

Adherence to MedDiet was assessed using the 17-item MedDiet validated questionnaire. Each item was related to a food habit and the compliance with food habits scored 1 for every item, otherwise scored 0; it ranged between 0 and 17 [39, 40].

#### CO_2_ emitted per kg of food

The amount of CO_2_ emitted per kg of consumed food per participant and day was calculated using a European database from 2016 that described kg of CO_2_ emitted per kg of food consumed. This database was based on life cycle assessment of recent studies and included agricultural production and processing steps (considering defaults for cooking, storing, and packing and letting transportation out of the calculations) [41]. Kilograms of CO_2_ emitted per consumed food were calculated by multiplying grams of each consumed food reported from the FFQ per kg of CO_2_ emitted per kg of each food from the database. The sum of all kilograms of CO_2_ emitted for all the products was done to determine the total emissions a day from diet. Once the CO_2_ emitted for each participant was known, an adjustment per 1 kg of food consumed was completed. The adjustment was done to consider the energy intake confounder. Depending on the individual needs, the dietary intake could be higher in terms of quantity meaning higher emissions, even when comparing diets based on the same products. Therefore, an adjustment per 1 kg of food product per person offers a better comparison between the emissions of the participants’ diets and avoids bias for people who could eat higher amounts due to their personal needs. Data was distributed in quartiles according to the amount of CO_2_ emissions: Quartile 1 (Q1) represented participants with the lowest emissions (≤ 2.01 kg CO_2_), quartile 2 (Q2) represented participants with low-moderate emissions (2.02–2.34 kg CO_2_), quartile 3 (Q3) represented participants with moderate-high emissions (2.35–2.79 kg CO_2)_ and quartile 4 (Q4) represented participants with the highest emissions (≥ 2.80 kg CO_2_). Q1 was established as the reference.

#### MetS assessment

MetS components were determined at baseline considering the following criteria [5]: high glucose levels (> 100 mg/dL), hypertension (> 130/85 mmHg), raised triglyceride levels (> 150 mg/dL), low high-density lipoprotein cholesterol levels (< 40 mg/dL in men; <50 mg/dL in women), and abdominal obesity (waist circumference of > 102 cm in men; >88 cm in women). The criteria are described as follows:

- High glucose blood level, or hyperglycemia, is a situation when the body is not able to produce enough insulin to transport glucose from blood to cells and it remains excessively in the bloodstream [42]. To assess glycemia levels, overnight fasting (at least 8 h) blood collections were analyzed in local laboratory using standard enzymatic methods.
- Hypertension is the high blood pressure exerted on the blood vessels [43]. Blood pressure was measured in seated position with a validated semi-automatic oscillometer (Omron HEM-705CP, Lake Forest, IL, USA). Three measures were taken after 5 min sitting at rest, waiting one minute between each take.
- Dyslipidemia is the altered blood lipid concentration. There are two MetS components related with dyslipidemia: High blood level of triglycerides, or hypertriglyceridemia, and low concentration of high-density lipoproteins (HDL), or low HDL-cholesterol. Overnight fasting blood collections were analyzed in local laboratory using standard enzymatic methods [44].
- Abdominal obesity or excessive accumulation of fat at abdomen [45] was assessed by measuring waist circumference two times using an anthropometric tape, halfway between the last rib and the iliac crest [46].

Each risk factor was assessed separately, as previously described, and its severity was calculated applying the MetSSS [11]. MetSSS was calculated using standard deviations and weight of Principal Component Analysis of all participants data from The Healthy Hearts (HH) study, which was a cross-sectional observational study aimed to establish age and sex cardiometabolic risk factors in an Australian population [47]. Its formula uses all MetS biomarker values for everyone (who are not using any prescribed medications), measuring the statistical distance from clinical thresholds for the MetS using the Mahalanobis Distance [48]. Specific steps for the MetSSS development are explained elsewhere [11]. MetSSS enables the conversion of the categorical variables, associated to MetS criteria, into a single continuous variable, facilitating the assessment not only of the presence of each risk factor but also the severity level associated with them. Instead of assessing each risk factor separately, MetSSS is used because it allows the assessment of the combined effects of having one or more aggregated risk factors, rather than solely considering their individual effects on a person’s state of health.

#### Other health variables

Information related to sociodemographic characteristics such as sex, age, and education levels were self-reported. Anthropometric measurements (weight, height, waist, and hip circumference) were obtained. The validated Minnesota-REGICOR short PA questionnaire [49–51] and the validated Spanish version of the Nurses’ Health Study questionnaire [52] were used to assess PA and sedentary behaviors respectively and data was showed in Metabolic Equivalents of Task (METs).

#### Statistics

Analyses were performed with the SPSS statistical software version 27.0 (SPPS Inc., Chicago, IL, USA). Data are shown as mean and standard deviation (SD). Prevalence was expressed in sample size and percentage. Differences among groups were tested with one-way ANOVA (followed by a post-hoc and Bonferroni analysis) for continuous variables and chi-squared test for categorical variables. Logistic regression was fitted to assess association between the MetS parameters and its severity, and the kg of emitted CO_2_. Odds Ratio (OR) value, crude and adjusted, and its interval was calculated. Quartiles were created to separate the sample according to the amount of emitted CO^2^ and Q1 was considered as the reference value. The first OR adjustment was made based on sociodemographic characteristics (sex, education level, age) and the second OR adjustment included sociodemographic characteristics (sex, education level and age) and adherence to the MedDiet. The first OR adjustment was done to consider sociodemographic characteristic effects on the sample. Sex, educational level, and age can alter metabolic syndrome severity outcomes [11]. The second OR adjustment added the MedDiet variable because it was seen inversely related with metabolic syndrome severity [10] and inversely related to CO_2_ emissions [53]. OR was calculated between each one of the items of the MetS, the MetS severity (low or high) and the quartiles of the amount of CO_2_ emitted in kg. Predictive margins were calculated with a 95% confidence interval (CI) and a linear prediction was created between quartiles of CO_2_ emissions and the MetSSS. Statistical significance was set at a two-tailed *p* value < 0.05. Our hypothesis was that a more sustainable diet, in terms of CO_2_ emissions, would be related to the risk of metabolic syndrome.

## Results

Table 1 shows sociodemographic characteristics of the sample. The relation between age, BMI, PA, sex, MetS parameters, and the severity of MetS according to the amount (kg) of CO_2_ emissions is described in Table 2. When comparing Q1 and Q4, age and BMI proved to be significant, with little changes across groups. Those individuals with higher levels of CO_2_ emissions were more likely to be men, younger and had lower BMI. Only glucose was significant (*p* = 0.039). Higher glycaemia levels (> 100 mg/dL) were identified in those with higher CO_2_ emissions (Q4: >2.80 kg CO_2_/day), with lower glycaemia levels observed in Q1 and Q2. The relationship between the severity of MetS and CO_2_ emissions was significant (*p* = 0.025). The percentage of people with higher MetS severity was higher in people with higher CO2 emissions (Q1: 42.7%; Q4: 47.7%) and the percentage of those with low severity was higher in groups with low CO2 emissions (Q1: 48.4%; Q4: 43.9%).

**Table 1:** Sociodemographic characteristics of the sample

	n (%)
Sex	
Men	3429 (51.6%)
Women	3218 (48.4%)
Highest school level completed	
Bachelor’s degree	858 (12.9%)
College School Technician	601 (9%)
Secondary School	1918 (28.9%)
Primary School	3270 (49.2%)
	mean (± SD)
Age (years)	64.9 (± 4.9)
Weight (Kg)	86.5 (± 12.9)
BMI (Kg/m^2^)	32.5 (± 3.4)
Energy intake (Kcal/day)	2365 (± 551)

Categorical variables are shown in sample size and percentage, and continuous variables by mean and standard deviation (SD)

*Abbreviation:* *BMI *Body Mass Index

**Table 2:** SES factors, physical activity and MetS criteria and severity according to CO_2_ emissions (quartiles)

	Q1 *n* = 1661	Q2 *n* = 1662	Q3 *n* = 1663	Q4 *n* = 1660	*p*-value
	Mean (SD)	Mean (SD)	Mean (SD)	Mean (SD)	
Age	65.2 (4.9) ^c^	65.1 (4.9)	64.8 (4.9)	64.8 (4.9) ^c^	0.020
BMI	32.4 (3.5) ^c^	32.5 (3.4)	32.5 (3.5)	32.7 (3.5) ^c^	0.039
Total PA (METs)	3328 (2789.6)	3019.7 (2995)	2349 (2552.4)	2785.9 (2699.3)	0.641
Light PA (METs)	788.2 (1064)	732.6 (918.8)	596.4 (740.9)	762.9 (980.7)	0.436
Moderate PA (METs)	1484.8 (2167.3)	1641.1 (2970.1)	1105.3 (2099.2)	1132.1 (1586.9)	0.294
Intense PA (METs)	1054.9 (1697.5)	645.9 (947.4)	647.2 (1275.5)	890.9 (1648.3)	0.231
	n (%)	n (%)	n (%)	n (%)	
Sex					
Men	788 (47.4)	824 (49.6)	884 (53.2)	932 (56.1)	< 0.001
Women	873 (52.6)	838 (50.4)	779 (46.8)	728 (43.9)	
MS Inclusion Criteria					
TG ≥ 150 mg/dL	939 (56.5)	891 (53.6)	940 (56.5)	927 (55.8)	0.276
Glycaemia ≥ 100 mg/dL	1210 (72.8)	1258 (75.7)	1270 (76.4)	1274 (76.7)	0.039
Hypertension ≥ 130/85mmHg	1516 (91.3)	1534 (92.3)	1535 (92.3)	1524 (91.8)	0.655
HDL cholesterol [< 40 mg/dL (M) /<50 mg/dL (W)]	707 (42.6)	689 (41.5)	737 (44.3)	709 (42.7)	0.419
Waist > 102 cm (M) > 88 cm (W)	1590 (95.7)	1600 (96.3)	1603 (96.4)	1591 (95.8)	0.712
MetS Severity^g^					
Low severity (≤ 3.31)	804 (48.4)	749 (45.1)	732 (44.0)	728 (43.9)	0.025
High severity (> 3.31)	709 (42.7)	759 (45.7)	765 (46.0)	791 (47.7)	

§Kg of CO2 per product= (Kg of the product from FFQ*Kg CO2 of the product in EU data base) / 1 kg of product. Adjustments for 1 kg of product were done later to be able to compare diets at the same level. Measurements were separated into four groups; Four groups were considered according to CO_2_ emissions: Q1: ≤2.01 kg CO_2_/day; Q2: 2.02–2.34 kg CO_2_/day; Q3: 2.35–2.80 kg CO_2_/day; Q4: >2.80 kg CO_2_/day. Difference in means between groups were tested by one-way ANOVA and Bonferroni’s post-hoc for age, BMI and physical activity. Differences in prevalence’s across groups were examined using χ2. Different letters indicate statistically significant differences between groups (a, b, c, d, e, f) according to Bonferroni’s post-hoc analysis

*Abbreviations:* *BMI *Body Mass Index, *MetS *Metabolic Syndrome, *TG *Triglycerides, *HDL *High Density lipoprotein

^g^MetS Severity is calculated with a Metabolic Syndrome Severity Score (MetSSS) [39]

The association between risk of MetS and the amount of CO_2_ emissions is shown in Table 3. Crude OR showed that glycaemia was significantly associated with Q2 (OR 1.16; 95%CI: 0.99–1.36), Q3 (OR 1.20; 95%CI: 1.03–1.41) and Q4 (OR 1.23; 95%CI: 1.05–1.44) in respect to Q1 (reference), meaning that people with higher glycaemia were more likely to have higher CO_2_ emissions. Results were also significant for glycaemia (*p* = 0.009) after adjustment in Q2 (OR 1.17; 95%CI: 1.00-1.37), Q3 (OR 1.23; 95%CI: 1.05–1.44) and Q4 (OR 1.29; 95%CI: 1.10–1.52). Crude and adjusted OR were also calculated for the severity of MetS and it shows how people with higher CO_2_ emissions were more likely to have higher severity of MetS compared to those with lower severity (Q2, OR 1.15; 95%CI: 1.00-1.33; Q3, OR 1.19; 95%CI: 1.03–1.37; Q4, OR 1.23; 95%CI: 1.07–1.42).

**Table 3:** Association between the risk of Metabolic Syndrome and CO_2_ emissions

		Q1 § *n* = 1661	Q2 § *n* = 1662	Q3 § *n* = 1663	Q4 § *n* = 1660	*p*-value
		OR (95% CI)	OR (95% CI)	OR (95% CI)	OR (95% CI)	
**MetS Criteria**						
TG ≥ 150 mg/dL	*Crude OR*	1.000 (Ref.)	0.89 (0.78–1.02)	1.00 (0.87–1.15)	0.97 (0.85–1.12)	0.277
	*OR adjusted 1*	1.000 (Ref.)	0.88 (0.88–1.01)	0.98 (0.85–1.12)	0.94 (0.82–1.08)	0.300
	*OR adjusted 2*	1.000 (Ref.)	0.88 (0.76–1.01)	0.96 (0.83–1.10)	0.91 (0.79–1.04)	0.240
Glucose < 100 mg/dL	*Crude OR*	1.000 (Ref.)	1.16 (0.99–1.36)	1.20 (1.03–1.41)	1.23 (1.05–1.44)	0.039
	*OR adjusted 1*	1.000 (Ref.)	1.16 (0.99–1.36)	1.20 (1.03–1.40)	1.22 (1.05–1.44)	0.045
	*OR adjusted 2*	1.000 (Ref.)	1.17 (1.00-1.37)	1.23 (1.05–1.44)	1.29 (1.10–1.52)	0.009
Hypertension ≥ 130/85mmHg	*Crude OR*	1.000 (Ref.)	1.15 (0.89–1.47)	1.15 (0.90–1.47)	1.07 (0.84–1.37)	0.655
	*OR adjusted 1*	1.000 (Ref.)	1.15 (0.90–1.48)	1.16 (0.91–1.50)	1.09 (0.85–1.40)	0.609
	*OR adjusted 2*	1.000 (Ref.)	1.15 (0.90–1.47)	1.16 (0.90–1.48)	1.07 (0.84–1.38)	0.635
HDL cholesterol	*Crude OR*	1.000 (Ref.)	0.96 (0.83–1.10)	1.07 (0.94–1.23)	1.00 (0.88–1.15)	0.419
< 40 mg/dL (M)	*OR adjusted 1*	1.000 (Ref.)	0.96 (0.84–1.10)	1.09 (0.95–1.25)	1.03 (0.90–1.18)	0.360
< 50 mg/dL (W)	*OR adjusted 2*	1.000 (Ref.)	0.96 (0.83–1.10)	1.07 (0.94–1.23)	1.01 (0.87–1.16)	0.435
Waist circumference	*Crude OR*	1.000 (Ref.)	1.15 (0.81–1.63)	1.19 (0.84–1.69)	1.03 (0.73–1.44)	0.712
> 102 cm (M)	*OR adjusted 1*	1.000 (Ref.)	1.21 (085-1.72)	1.33 (0.93–1.89)	1.21 (0.86–1.71)	0.455
> 88 cm (W)	*OR adjusted 2*	1.000 (Ref.)	1.21 (0.85–1.72)	1.33 (0.93–1.90)	1.21 (0.87–1.72)	0.455
MetS Severity^*^	*Crude OR*	1.000 (Ref.)	1.15 (1.00-1.33)	1.19 (1.03–1.37)	1.23 (1.07–1.42)	0.025
	*OR adjusted 1*	1.000 (Ref.)	1.18 (1.02–1.36)	1.25 (1.08–1.44)	1.33 (1.16–1.55)	0.001
	*OR adjusted 2*	1.000 (Ref.)	1.18 (1.02–1.36)	1.23 (1.06–1.42)	1.30 (1.13–1.51)	0.003

§Kg of CO2 per product= (Kg of the product from FFQ*Kg CO_2_ of the product in EU data base) / 1 kg of product. Adjustments for 1 kg of product were done later to be able to compare diets at the same level. Measurements were separated into four groups; Four groups were considered according to CO_2_ emissions: Q1: ≤2.01 kg CO_2_/day; Q2: 2.02–2.34 kg CO_2_/day; Q3: 2.35–2.80 kg CO_2_/day; Q4: >2.80 kg CO_2_/day

*Abbreviations:* *MetS *Metabolic Syndrome, *TG *Triglycerides, *HDL *High Density lipoprotein, *OR *Odds Ratio, *OR adjusted 1*: Odds Ratio adjusted by sociodemographic characteristics (sex, education level, age). *OR adjusted 2*: Odds Ratio adjusted by sociodemographic characteristics (sex, education level, age) and adherence to the Mediterranean Diet

^*^MetS Severity is calculated with a Metabolic Syndrome Severity Score (MetSSS) [39]

**Fig. 2:**
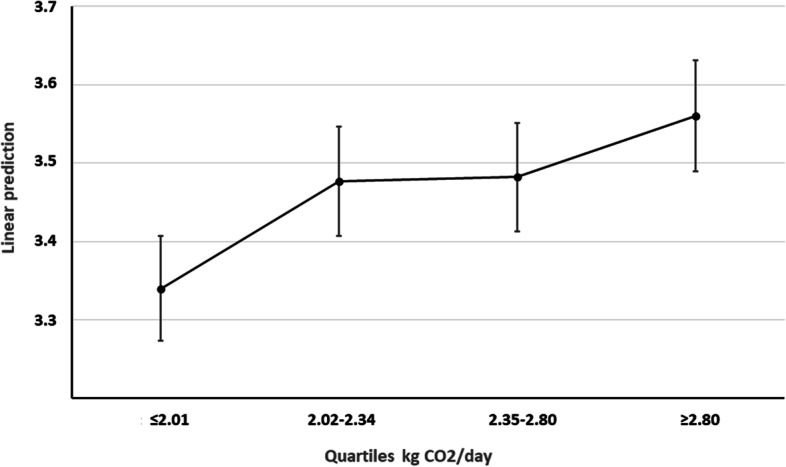
Predictive margins between kg CO_2_ quartiles and MetS severity with 95%CI

Figure 2 shows a linear prediction between the amounts of CO_2_ emitted (separated in quartiles) and the MetS severity score. A direct relation can be seen in the graph showing that as CO2 emissions increase, the level of severity of the MetS increases as well. The greater difference may be noticed in the graph when comparing Q1 and Q4.

## Discussion

The current study showed the relation between MetS and CO_2_ emissions. Following a diet which emitted less CO_2_ to the atmosphere appeared to be related with lower MetS severity. Especially, people with high glycaemia were highly related to high CO_2_ emissions.

A systematic review published in 2019 showed that most of the studies assessing sustainability aimed to reduce GHGs such as CO_2_ [24] which is the greenhouse gas used for the calculations in this paper. The major published papers tended to relate environmental effect with the type of food consumed, occasionally analyzing the environmental impact of the entire diet [54]. The vegetarian diet has received attention not just in terms of sustainability but also in terms of health. It is based on products that have a lesser environmental effect and implies a reduction in high GHG emitting foods which are principally meat and animal products. It is a plant-based diet that could store carbon into the soil due to photosynthetic effects and release oxygen reducing the overall amount of atmospheric CO_2_. Moreover, individuals who followed these diets appeared to have lower incidence of several non-communicable diseases [55]. When comparing different diets in terms of specific food choices, high meat-eaters result to be those who eat foods with a higher carbon footprint. Reductions in meat consumption, particularly red and processed meat, dairy products, and others like sweets, savory snacks, white bread, and beverages, may lead to optimized diets in terms of sustainability. A reduction in energy intake has also been identified as a key component in lowering GHGs [56, 57].

The research on relating GHGs and MetS is limited. However, relevant studies relating GHGs, and healthy or unhealthy situations have been found. The Lancet Commission established in 2019 that ‘*The food we eat and how we produce it will determine the health of people and planet, and major changes must be made to avoid both reduced life expectancy and continued environmental degradation*’ [4]. Accordingly, several institutions like the World Health Organization had policies to emphasize the importance of change to a sustainable lifestyle, being sustainable healthy diets a starting point [58]. The Paris agreement is another known consensus aiming to limit global warming below 2ºC and closer to 1.5ºC. To achieve it, the adhered countries should limit their GHGs to a predetermined level [4].

One study assessing health and environmental impacts of the food-based dietary guidelines from 85 different countries compared the strategies done with the WHO and the EAT-Lancet Commission recommendations [59]. A reduction of premature mortality (15%) and a reduction in GHGs (13%) were found when the national food based dietary guidelines were adopted. The biggest improvement was following the EAT-Lancet Commission recommendations; 34% lower premature mortality, more than three times greater reductions in GHGs and, in general, greater achievement of the global targets. Even though these differences are not causal, they are associated with better dietary choices [59].

Non-communicable diseases were mentioned in some studies as being related to sustainability [60, 61]. Standard dietary guidelines were compared to a 2050 reference scenario which revealed that switching to plant-based diets and reducing animal-source foods would prevent 5.1 million deaths per year and preserve 79 million years of life. Moreover, if a vegetarian or vegan diet is selected, those figures will rise. A reduced consumption of meat and higher consumption of fruits and vegetables would lead to a reduction in mortality numbers and a 19–30% lower prevalence of being overweight or obese, associated with a limited energy intake [60]. Adopting a diet scenario in line with health and sustainable recommendations resulted in 45–47% prevented deaths from reduced coronary heart disease, 26% from stroke, 16–18% from cancer and 10–12% from T2DM [60].

Given MetS is specifically a risk factor of those diseases, a low CO_2_ emissions-diet could be a safe and useful strategy. It was pointed out that the adoption of different diets like the Mediterranean, pescatarian, and vegetarian would reduce emissions by 30%, 45% and 55% respectively [61]. Depending on the diet followed, the risk of type 2 diabetes mellitus, cancer, coronary mortality, or all-cause mortality could be reduced [62, 63]. The current study has also shown that a low CO_2_ emissions-diet, which could be related to a lower risk of having high glycaemia levels in a Mediterranean population.

Another study, where non-communicable diseases were related to several diet scenarios, showed that the current Swiss diet appeared to be the most beneficial in terms of health and sustainability; on the contrary, a meat-oriented diet could result in adverse health outcomes, a higher environmental footprint, an increase in daily food expenditure and a lack of some essential nutrients [64]. An ecological study from United States showed associations between CO_2_ emissions and the prevalence of obesity and diabetes [65], which agrees with the current study, although those associations were weaker after some adjustments.

Food dietary choices influence MetSSS and CO_2_ emissions, but they can also be influenced by social, economic, commercial, and political factors. Metabolic syndrome is classified as a non-communicable disease and, even if it started being more prevalent in developed countries, it spread due to presence of the western lifestyle around the world [12]. Moreover, healthy foods have become more expensive than unhealthy ones, having ultra-processed products as an example [66], driving people with fewer resources to purchase them [67] and affecting planet and people’s health. Commercial determinants of health can also be affecting both dimensions, including various sectors such as the food and beverage industry, tobacco industry, alcohol industry, pharmaceutical industry, and advertising and marketing industries. These sectors often employ strategies to promote their products, maximize profits and achieve economic growth, having significant implications for public health and the environment [68]. The regulation of these determinants needs to be addressed by political systems, policies, and governance structures, being the political factor also a health-environment determinant itself. There is a need of developing policies focused on the sustainable development goals [69] and integrating climate change and health systems [70, 71].

It is true that there is already some research on the relation of what we eat, the environment and some specific health conditions, but very few information is found when relating diet, environment, and MetS. This new study on this subject showed how being more conscious and modifying our eating habits and could be related both with the planet’s health and the people’s quality of life.

### Strengths and limitations of the study

The first strength is that the current study contributes to the very limited evidence relating sustainability, in terms of CO_2_ emissions, and MetS. The second strength is the large sample size used. Once the CO_2_ calculations were done for each participant, an adjustment per 1 kg of food product was done. This is a strength because it avoids the effect of the energy intake confounder. The amount in grams of food consumed is closely related to individual caloric requirements. In other words, high energy requirements are met by high amounts of food, and accordingly low requirements relate to lower amounts of food. Quantity of food is directly related to CO_2_ emissions. To avoid this bias, an adjustment per one kg of food product was performed. Calculating only the parameter of CO_2_ emissions for assessing the sustainability of a diet allows the impact to be observed independently from other parameters.

The current study has also limitations. The environmental impact is just calculated in terms of CO_2_ emissions while other studies have considered water, land, or energy use as well as marine eutrophication, atmospheric acidification, and nitrogen or phosphorous release [55, 72]. The database used to calculate CO_2_ emissions is from 2016, which is the data collected in the current paper. This makes sense as data analyzed according to the situation at current time. Moreover, a while has passed from 2016 and some data might not be the latest available. MetSSS was originally created using data from an Australian population which is another limitation, since our population is Spanish. The fact that the population studied were 55 to 75 years old, limits the possibility to apply the current findings on younger populations. Finally, causal effects cannot be set since the study presents a cross-sectional design.

## Conclusions

CO_2_ emission could be related with the risk of having a more severe MetS and with glycaemia. Following a diet which emitted less CO_2_ to the atmosphere could be helpful for those participants with high MetS severity. Further research is needed to assess the relation between sustainability and some specific non-communicable diseases, to find more ways to reduce the mortality and morbidity they are causing worldwide.

## Data Availability

Data described in the manuscript, code book, and analytic code will be made available upon request pending application and approval of the PREDIMED-Plus Steering Committee. There are restrictions on the availability of data for the PREDIMED-Plus trial, due to the signed consent agreements around data sharing, which only allow access to external researchers for studies following the project purposes. Requestors wishing to access the PREDIMED-Plus trial data used in this study can make a request to the PREDIMED-Plus trial Steering Committee chair: jordi.salas@urv.cat. The request will then be passed to members of the PREDIMED-Plus Steering Committee for deliberation.

## Abbreviations

BMI: Body mass index
CO_2_: Carbon Dioxide
FFQ: Food frequency questionnaire
GHGs: Greenhouse gas emissions
HDL: High Density lipoprotein
MedDiet: The Mediterranean Diet
MetS: Metabolic Syndrome
MetSS: Metabolic Syndrome Severity
MetSSS: Metabolic Syndrome Severity Score
OR: Odds Ratio
Q1: Quartile 1
Q2: Quartile 2
Q3: Quartile 3
Q4: Quartile 4
SD: Standard deviations
TG: Triglycerides
T2DM: Type 2 diabetes mellitus

## References

[1] Moreno Rodríguez JM. (coordinator). A Preliminary Assessment of the Impacts in Spain due to the Effects of Climate Change. ECCE project, final report. Madrid: Ministerio de Medio Ambiente, 765 p., 2005.

[2] Pachauri RK, Allen MR, Barros VR, Broome J, Cramer W, Christ R et al. Climate Change 2014: Synthesis Report. Contribution of Working Groups I, II and III to the Fifth Assessment Report of the Intergovernmental Panel on Climate Change. Geneva, Switzerland. R. Pachauri and L. Meyer, editors, 2015. Available from: https://www.ipcc.ch/site/assets/uploads/2018/05/SYR_AR5_FINAL_full_wcover.pdf. Accessed 19 Nov 2021.

[3] United Nations (UN). UN News. Global perspective Human stories; 09 Mar 2019. Food systems account for over one-third of global greenhouse gas emissions. Available from: https://news.un.org/en/story/2021/03/1086822. Accessed 19 Nov 2021.

[4] Willett W, Rockström J, Loken B, Springmann M, Lang T, Vermeulen S (2019). Food in the Anthropocene: the EAT–Lancet Commission on healthy diets from sustainable food systems. Lancet.

[5] Alberti KG, Eckel RH, Grundy SM, Zimmet PZ, Cleeman JI, Donato KA et al. Harmonizing the metabolic syndrome: a joint interim statement of the International Diabetes Federation Task Force on Epidemiology and Prevention; National Heart, Lung, and Blood Institute; American Heart Association; World Heart Federation; International Atherosclerosis Society; and International Association for the Study of Obesity. Circulation. 2009;120. 1640–1645. 10.1161/CIRCULATIONAHA.109.192644 19805654

[6] Engin A (2017). The definition and prevalence of obesity and metabolic syndrome. Adv Exp Med Biol.

[7] Sherling DH, Perumareddi P, Hennekens CH (2017). Metabolic syndrome: clinical and policy implications of the New Silent Killer. J Cardiovasc Pharmacol Therap.

[8] Moreira GC, Cipullo JP, Souza-Ciorlia LA, Bernardi-Cesarino C, Vilela-Martin JF. Prevalence of metabolic syndrome: Association with risk factors and cardiovascular complications in an urban population. PLoS ONE. 2014;9:e105056. 10.1371/journal.pone.0105056 PMC4152120 25180496

[9] Instituto Nacional de Estadística. INE. Defunciones según causa de muerte. Avance enero-mayo 2020. Available from: https://www.ine.es/mapas/svg/indicadoresDefuncionCausa.htm. Accessed 19 Nov 2021.

[10] O’Neill S, O’Driscoll L (2015). Metabolic syndrome: a closer look at the growing epidemic and its associated pathologies. Obes Rev.

[11] Wiley JF, Carrington MJ (2016). A metabolic syndrome severity score: a tool to quantify cardio-metabolic risk factors. Prev Med.

[12] Saklayen MG (2018). The global epidemic of the metabolic syndrome. Curr Hypertens Rep.

[13] Renninger M, Hansen BH, Steene-Johannessen J, Kriemler S, Froberg K, Northstone K (2020). Associations between accelerometry measured physical activity and sedentary time and the metabolic syndrome: a meta-analysis of more than 6000 children and adolescents. Pediatr Obes.

[14] Gallardo-Alfaro L, Bibiloni MM, Mascaró CM, Montemayor S, Ruiz-Canela M, Salas-Salvado J (2020). Leisure-time physical activity, sedentary behaviour and diet quality are associated with metabolic syndrome severity: the PREDIMED-plus study. Nutrients.

[15] Earnest CP, Johannsen NM, Swift DL, Gillison FB, Mikus CR, Lucia A (2014). Aerobic and strength training in concomitant metabolic syndrome and type 2 diabetes. Med Sci Sports Exerc.

[16] Tai HC, Tzeng IS, Liang YC, Liao HH, Su CH, Kung WM (2019). Interventional effects of weight-loss policy in a healthy city among participants with metabolic syndrome. Int J Environ Res Public Health.

[17] Dieli-Conwright CM, Courneya KS, Demark-Wahnefried W, Sami N, Lee K, Buchanan TA et al. Effects of Aerobic and Resistance Exercise on Metabolic Syndrome, Sarcopenic Obesity, and Circulating Biomarkers in Overweight or Obese Survivors of Breast Cancer: A Randomized Controlled Trial [published correction appears in J Clin Oncol. 2020 Apr 20;38(12):1370] [published correction appears in J Clin Oncol. 2020 Jun 20;38(18):2115]. J Clin Oncol. 2018;36:875–883. 10.1200/JCO.2017.75.7526 PMC5858524 29356607

[18] Julibert A, Bibiloni MM, Mateos D, Angullo E, Tur JA. Dietary fat intake and metabolic syndrome in older adults. Nutrients. 2019;11:1901. 10.3390/nu11081901 PMC6723812 31416272

[19] Wilkinson MJ, Manoogian ENC, Zadourian A, Lo H, Fakhouri S, Shoghi A (2020). Ten-hour time-restricted eating reduces weight, blood pressure, and atherogenic lipids in patients with metabolic syndrome. Cell Metab.

[20] Campos-Nonato I, Hernandez L, Barquera S (2017). Effect of a high-protein Diet versus Standard-Protein Diet on Weight loss and biomarkers of metabolic syndrome: a Randomized Clinical Trial. Obes Facts.

[21] Velázquez-López L, Santiago-Díaz G, Nava-Hernández J, Muñoz-Torres AV, Medina-Bravo P, Torres-Tamayo M (2014). Mediterranean-style diet reduces metabolic syndrome components in obese children and adolescents with obesity. BMC Pediatr.

[22] Sayón-Orea C, Razquin C, Bulló M, Corella D, Fitó M, Romaguera D (2019). Effect of a nutritional and behavioral intervention on Energy-Reduced Mediterranean Diet adherence among patients with metabolic syndrome: interim analysis of the PREDIMED-Plus Randomized Clinical Trial. JAMA.

[23] Fortin A, Rabasa-Lhoret R, Lemieux S, Labonté ME, Gingras V (2018). Comparison of a Mediterranean to a low-fat diet intervention in adults with type 1 diabetes and metabolic syndrome: a 6–month randomized trial. Nutr Metab Cardiovasc Dis.

[24] Babio N, Toledo E, Estruch R, Ros E, Martínez-González MA, Castañer O (2014). Mediterranean diets and metabolic syndrome status in the PREDIMED randomized trial. CMAJ.

[25] Wilson N, Cleghorn CL, Cobiac LJ, Mizdrak A, Nghiem N (2019). Achieving healthy and sustainable diets: a review of the results of recent Mathematical optimization studies. Adv Nutr.

[26] Riediger ND, Clara I. Prevalence of metabolic syndrome in the Canadian adult population. CMAJ. 2011;183:E1127-E1134. 10.1503/cmaj.110070 PMC3193129 21911558

[27] Fernández-Bergés D, Cabrera de León A, Sanz H, Elosua R, Guembe MJ, Alzamora M (2012). Metabolic syndrome in Spain: prevalence and coronary risk associated with harmonized definition and WHO proposal. DARIOS study. Rev Esp Cardiol (Engl Ed).

[28] Beltrán-Sánchez H, Harhay MO, Harhay MM, McElligott S (2013). Prevalence and Trends of metabolic syndrome in the adult U.S. Population, 1999–2010. J Am Coll Cardiol.

[29] Salas-Salvadó J, Díaz-López A, Ruiz-Canela M, Basora J, Fitó M, Corella D (2019). Effect of a lifestyle intervention program with energy-restricted Mediterranean diet and exercise on weight loss and cardiovascular risk factors: one-year results of the PREDIMED-Plus trial. Diabetes Care.

[30] IEA. Global CO2 emissions in 2019. Available from: https://www.iea.org/articles/global-co2-emissions-in-2019. Accessed 19 Nov 2021.

[31] Fresán U, Martínez-Gonzalez MA, Sabaté J, Bes-Rastrollo M (2018). The Mediterranean diet, an environmentally friendly option: evidence from the Seguimiento Universidad de Navarra (SUN) cohort. Public Health Nutr.

[32] Burlingame B, Dernini S. Sustainable Diets and Biodiversity: Directions and Solutions for Policy, Research and Action. Proceedings of the International Scientific Symposium ‘Biodiversity and Sustainable Diets United Against Hunger’. Rome: FAO Headquarters; 3–5 November 2010 [accessed September 2022]. Available: http://www.fao.org/docrep/016/i3004e/i3004e.pdf.

[33] Martínez-González MA, Buil-Cosiales P, Corella D, Bulló M, Fitó M, Vioque J (2019). Cohort profile: design and methods of the PREDIMED-Plus randomized trial. Int J Epidemiol.

[34] Fernández-Ballart JD, Piñol JL, Zazpe I, Corella D, Carrasco P, Toledo E (2010). Relative validity of a semi-quantitative food-frequency questionnaire in an elderly Mediterranean population of Spain. Br J Nutr.

[35] Martin-Moreno JM, Boyle P, Gorgojo L, Maisonneuve P, Fernandez-Rodriguez JC, Salvini S (1993). Development and validation of a food frequency questionnaire in Spain. Int J Epidemiol.

[36] de la Fuente-Arrillaga C, Ruiz ZV, Bes-Rastrollo M, Sampson L, Martinez-González MA (2010). Reproducibility of an FFQ validated in Spain. Public Health Nutr.

[37] Lupiañez-Barbero A, González Blanco C, de Leiva Hidalgo A (2018). Spanish food composition tables and databases: need for a gold standard for healthcare professionals. Tablas y bases de datos de composición de alimentos españolas: necesidad de un referente para los profesionales de la salud. Endocrinol Diabetes Nutr (Engl Ed).

[38] Mataix J, Mañas M, Llopis J, Martínez de Victoria E, Juan J, Borregón A (2013). Tablas de Composición de Alimentos (Spanish Food Composition tables).

[39] Álvarez-Álvarez I, Martínez-González M, Sánchez-Tainta A, Corella D, Díaz-López A, Fitó M (2019). Adherence to an energy-restricted Mediterranean Diet score and prevalence of cardiovascular risk factors in the PREDIMED-Plus: a cross-sectional study. Rev Esp Cardiol (Engl Ed).

[40] Galilea-Zabalza I, Buil-Cosiales P, Salas-Salvadó J, Toledo E, Ortega-Azorín C, Díez-Espino J et al. Mediterranean diet and quality of life: Baseline cross-sectional analysis of the PREDIMED-PLUS trial. PLoS ONE 2018, 13, e0198974. 10.1371/journal.pone.0198974 PMC6005498 29912978

[41] Hartikainen H, Pulkkinen H. Summary of the chosen methodologies and practices to produce GHGE-estimates for an average European diet. 2016 Available from: http://luke.juvenesprint.fi. Accessed 19 Nov2021.

[42] American Diabetes Association. 5. Lifestyle Management: Standards of Medical Care in Diabetes-2019. Diabetes Care. 2019;42(Suppl 1):S46-S60. 10.2337/dc19-S005 30559231

[43] Mancia G, Fagard R, Narkiewicz K, Redón J, Zanchetti A, Böhm M (2013). 2013 ESH/ESC Guidelines for the management of arterial hypertension: the Task Force for the management of arterial hypertension of the European Society of Hypertension (ESH) and of the European Society of Cardiology (ESC). J Hypertens.

[44] Lozano JA (2005). Dislipidemias. Pautas para su abordaje terapéutico. Offarm.

[45] World Health Organization; 9. June 2021. Obesity and overweight. Available from: https://www.who.int/es/news-room/factsheets/detail/obesity-and-overweight. Accessed 12 June 2023.

[46] Martínez-González MA, Buil-Cosiales P, Corella D, Bulló M, Fitó M, Vioque J (2019). Cohort profile: design and methods of the PREDIMED-Plus randomized trial. Int J Epidemiol.

[47] Carrington MJ, Jennings GL, Clark RA, Stewart S (2012). Assessing cardiovascular risk in regional areas: the healthy Hearts - Beyond City limits program. BMC Health Serv.

[48] Mahalanobis PC (1936). On the generalized distance in statistics. Proc Natl Inst Sci.

[49] Elosua R, Garcia M, Aguilar A, Molina L, Covas MI, Marrugat J (2000). Validation of the Minnesota Leisure Time Physical Activity Questionnaire in Spanish Women. Med Sci Sports Exerc.

[50] Elosua R, Marrugat J, Molina L, Pons S, Pujol E (1994). Validation of the Minnesota Leisure Time Physical Activity Questionnaire in spanish men. Am J Epidemiol.

[51] Molina L, Sarmiento M, Peñafiel J, Donaire D, Garcia-Aymerich J, Gomez M (2017). Validation of the Regicor Short Physical Activity Questionnaire for the Adult Population. PLoS ONE.

[52] Martínez-González MA, López-Fontana C, Varo JJ, Sánchez-Villegas A, Martinez JA (2005). Validation of the spanish version of the physical activity questionnaire used in the Nurses’ Health Study and the Health Professionals’ follow-up study. Public Health Nutr.

[53] García S, Bouzas C, Mateos D, Pastor R, Álvarez L, Rubín M (2023). Carbon dioxide (CO_2_) emissions and adherence to Mediterranean diet in an adult population: the Mediterranean diet index as a pollution level index. Environ Health.

[54] Behrens P, Kiefte-De Jong JC, Bosker T, Rodrigues JFD, de Koning A, Tukker A (2017). Evaluating the environmental impacts of dietary recommendations. Proc Natl Acad Sci USA.

[55] Grosso G, Fresán U, Bes-Rastrollo M, Marventano S, Galvano F (2020). Environmental impact of dietary choices: role of the Mediterranean and other dietary patterns in an italian cohort. Int J Environ Res Public Health.

[56] Perignon M, Vieux F, Soler LG, Masset G, Darmon N (2017). Improving diet sustainability through evolution of food choices: review of epidemiological studies on the environmental impact of diets. Nutr Rev.

[57] Springmann M, Wiebe K, Mason-D’Croz D, Sulser TB, Rayner M, Scarborough P (2018). Health and nutritional aspects of sustainable diet strategies and their association with environmental impacts: a global modelling analysis with country-level detail. Lancet Planet Health.

[58] World Health Organization, Food and Agriculture Organization of the United Nations; 29. October 2019. Sustainable healthy diets: guiding principles. Available from: https://www.who.int/publications/i/item/9789241516648. Accessed 19 Nov 2021.

[59] Springmann M, Spajic L, Clark MA, Poore J, Herforth A, Webb P (2020). The healthiness and sustainability of national and global food based dietary guidelines: modelling study. BMJ.

[60] Springmann M, Charles H, Godfray J, Rayner M, Scarborough P. Analysis and valuation of the health and climate change co-benefits of dietary change. Proc Natl Acad Sci USA. 2016;113:4146–4151. 10.1073/pnas.1523119113 PMC4839446 27001851

[61] Tilman D, Clark M (2014). Global diets link environmental sustainability and human health. Nature.

[62] Esposito K, Chiodini P, Colao A, Lenzi A, Giugliano D (2012). Metabolic syndrome and risk of cancer: a systematic review and meta-analysis. Diabetes Care.

[63] Moreira GC, Cipullo JP, Ciorlia LAS, Cesarino CB, Vilela-Martin JF. Prevalence of metabolic syndrome: Association with risk factors and cardiovascular complications in an urban population. PLoS ONE 2014, 9, e105056. 10.1371/journal.pone.0105056 PMC4152120 25180496

[64] Chen C, Chaudhary A, Mathys A (2019). Dietary change scenarios and implications for environmental, nutrition, human health and economic dimensions of food sustainability. Nutrients.

[65] Zheutlin AR, Adar SD, Park SK (2014). Carbon dioxide emissions and change in prevalence of obesity and diabetes in the United States: an ecological study. Environ Int.

[66] García S, Pastor R, Monserrat-Mesquida M, Álvarez-Álvarez L, Rubín-García M, Martínez-González MA et al. Ultra-processed foods consumption as a promoting factor of greenhouse gas emissions, water, energy, and land use: A longitudinal assessment. Sci Total Environ. 2023;891:164417. 10.1016/j.scitotenv.2023.164417 37236477

[67] Gupta S, Hawk T, Aggarwal A, Drewnowski A (2019). Characterizing Ultra-Processed Foods by Energy Density, Nutrient Density, and cost. Front Nutr.

[68] World Health Organization; 21. March 2023. Commercial determinants of health. Available from: https://www.who.int/news-room/fact-sheets/detail/commercial-determinants-of-health. Accessed 12 June 2023.

[69] World Health Organization. ; 2023. THE 17 GOALS. Available from: https://sdgs.un.org/goals. Accessed 12 June 2023.

[70] Anstey MH (2013). Climate change and health–what’s the problem?. Global Health.

[71] Hutton G (2011). The economics of health and climate change: key evidence for decision making. Global Health.

[72] Fresán U, Martínez-González MA, Sabaté J, Bes-Rastrollo M. Global sustainability (health, environment and monetary costs) of three dietary patterns: results from a spanish cohort (the SUN project). BMJ Open 2019,9, e021541. 10.1136/bmjopen-2018-021541 PMC6398639 30796113

